# Measurement of vertebral rotation in adolescent idiopathic scoliosis with low-dose CT in prone position - method description and reliability analysis

**DOI:** 10.1186/1748-7161-5-4

**Published:** 2010-02-23

**Authors:** Kasim Abul-Kasim, Magnus K Karlsson, Ralph Hasserius, Acke Ohlin

**Affiliations:** 1 Faculty of Medicine, Lund University, Division of Neuroradiology, Diagnostic Centre for Imaging and Functional Medicine, Skåne University Hospital, 20502 Malmö, Sweden; 2 Clinical and Molecular Osteoporosis Unit, Department of Clinical Sciences, Lund University, Sweden; 3 Department of Orthopaedics, Skåne University Hospital, 20502 Malmö, Sweden

## Abstract

**Background:**

To our knowledge there is no report in the literature on measurements of vertebral rotation with low-dose computed tomography (CT) in prone position.

**Aims:**

To describe and test the reliability of this new method, compare it with other methods in use and evaluate the influence of body position on the degree of vertebral rotation measured by different radiological methods.

**Study design:**

Retrospective study.

**Methods:**

25 consecutive patients with adolescent idiopathic scoliosis scheduled for surgery (17 girls, 8 boys) aged 15 ± 2 years (mean ± SD) were included in the analysis of this study. The degree of the vertebral rotation was in all patients measured according to the method of Perdriolle on standing plain radiographs and on supine CT scanogram, and according to the method of Aaro and Dahlborn on axial CT images in prone position and on magnetic resonance imaging (MRI) in supine position. The measurements were done by one neuroradiologist at two different occasions. Bland and Altman statistical approach was used in the reliability assessment.

**Results:**

The reliability of measuring vertebral rotation by axial CT images in prone position was almost perfect with an intraclass correlation coefficient of 0.95, a random error of the intraobserver differences of 2.3°, a repeatability coefficient of 3.2° and a coefficient of variation of 18.4%. Corresponding values for measurements on CT scanogram were 0.83, 5.1°, 7.2°, and 32.8%, respectively, indicating lower reliability of the latter modality and method. The degree of vertebral rotation measured on standing plain radiographs, prone CT scanogram, axial images on CT in prone position and on MRI in supine position were 25.7 ± 9.8°, 21.9 ± 8.3°, 17.4 ± 7.1°, and 16.1 ± 6.5°, respectively. The vertebral rotation measured on axial CT images in prone position was in average 7.5% larger than that measured on axial MRI in supine position.

**Conclusions:**

This study has shown that measurements of vertebral rotation in prone position were more reliable on axial CT images than on CT scanogram. The measurement of vertebral rotation on CT (corrected to the pelvic tilt) in prone position imposes lower impact of the recumbent position on the vertebral rotation than did MRI in supine position. However, the magnitude of differences is of doubtful clinical significance.

## Background

Estimation of the degree of vertebral rotation before posterior scoliosis corrective surgery using the method introduced by Suk in 1994 [1] (nowadays known as "all pedicle screw construct) helps to determine the transverse screw angle (TSA), which in turn determines the screw tract. Furthermore, knowledge of the degree of vertebral rotation is an indicator of curve progression and subsequently a predictive factor for the overall prognosis of this spinal deformity [2,3]. Therefore, a preoperative measurement of the degree of vertebral rotation provides the surgeon with information necessary for correct insertion of the pedicle screws at different vertebral levels. With regard to the measurement of vertebral rotation, the choice of the radiological modality, the method of measurement and the patient's position have been a matter of debate. The two most widely used radiological modalities are plain radiography and CT. The major drawback of CT is the high radiation dose but recently CT with low radiation dose has been shown to be a reliable method in the perioperative work-up of scoliosis [4]. MRI is another modality that can be used for these purposes [5]. However, the major disadvantages of MRI are less availability, longer examination time and the need for multiple acquisitions of axial sequences to cover the region of interest, often the whole thoracic and lumbar region. There are several methods used to measure the degree of vertebral rotation on plain radiographs, e.g. the methods proposed by Perdriolle and Vidal, Nash and Moe, Drerup, and Stokes [2,6-8], of which the method of Perdriolle is probably the most widely used one. Similarly, different methods have been used for measurements of the degree of vertebral rotation on CT, e.g. the methods proposed by Aaro and Dahlborn, Ho and Krismer [9-11], at present the method of Aaro and Dahlborn is the most widely used. The accuracy of the measurement of vertebral rotation with Perdriolle torsionmeter on plain radiographs varies widely in literature with some studies showing low inter- and intraobserver agreement [12], whereas other studies showed that Perdriolle torsionmeter is a reliable instrument to measure the degree of vertebral rotation [13,14].

Spontaneous correction of vertebral rotation occurs in recumbent position, in the literature reported to vary between 19 and 31% [15-17]. This makes it difficult to compare the degree of vertebral rotation measured on standing plain radiographs using the Perdriolle method with that measured on CT in recumbent position using the Aaro and Dahlborn method. However, new CT scanners enable acquisition of good quality scanogram corresponding to a radiograph in recumbent position. This enabled Yazici et al. [17] to conclude that the Perdriolle method was as accurate as the Aaro and Dahlborn method in determination of the degree of vertebral rotation when directly comparing the two methods. However, the statistical analysis used in that study has previously being criticized and considered as misleading, when assessing the agreement between two clinical measurements [18]. Automatic measurements of vertebral rotation was showed to be comparable with manual measurements according to the method of Aaro and Dahlborn and with the advantage of avoiding intra- or interobserver error due to landmark point selection [19].

With this background, the first aim of this study was to describe a previously not reported method of using low-dose CT in prone position in the assessment of the degree of vertebral rotation. The second aim was to study the reliability of this method and compare it with other radiological methods of measurement of vertebral rotation. As posterior corrective surgery is performed in prone position, we sought to perform CT examinations in prone position to provide figures of vertebral rotation measured in a body position identical to that the surgeons usually are faced with on the operating table. The final aim of the study was to evaluate the magnitude of the spontaneous correction of vertebral rotation achieved by the recumbent prone and supine position.

## Methods

We performed retrospective analysis of consecutively collected patients, 17 girls and 8 boys with mean age 15 ± 2 years (median 15 and range 11-20 years), scheduled for scoliosis surgery. The patients included in the analysis of this study were patients with adolescent idiopathic scoliosis (AIS) who underwent plain radiography, CT and MRI of the spine within a maximal interval of 2 months between theses examinations. All plain radiographs were performed at the same day as CT to enable the measurement of the deformity in coronal and sagittal planes. The apical vertebral rotation was measured at the following levels T7 (n = 2), T8 (n = 5), T9 (n = 7), T10 (n = 2), T11 (n = 1), T12 (n = 3), L1 (n = 3), and L2 (n = 2).

### Low-dose CT

All CTs were done in prone position on a 16-slice CT scanner (SOMATOM Sensation 16, Siemens AG, Forchheim, Germany) according to CT protocol with low radiation dose [4] covering the thoracic and lumbar spine (average 15 vertebral bodies). The scan parameters were the following: Slice collimation 16 × 0.75 mm, rotation time 0.75 second, pitch 1.5, tube voltage 80 kV, and quality reference for the effective tube current-time product 25 mAs. The dose reduction system (DRS) (CareDose 4D, Siemens AG, Forchheim, Germany) available in the scanner was automatically activated and contributed to reduction of effective tube current-time product to 19 mAs. Reconstructed slices with 3 mm thickness and 3 mm increment were obtained. The slice collimation of 0.75 mm allowed obtaining 1 mm thick reformatted axial images (1 mm increment) with both soft tissue algorithm and skeletal algorithm as well as 2 mm thick coronal and sagittal reformatted images. Furthermore, four sequential slices at the level of femoral heads were also obtained with tube voltage 80 kV and quality reference for the effective tube current-time product 25 mAs.

The use of low-dose CT in the perioperative work-up of patients with scoliosis was approved by the regional radiation protection committee.

### Measurement of vertebral rotation

**Figure 1:**
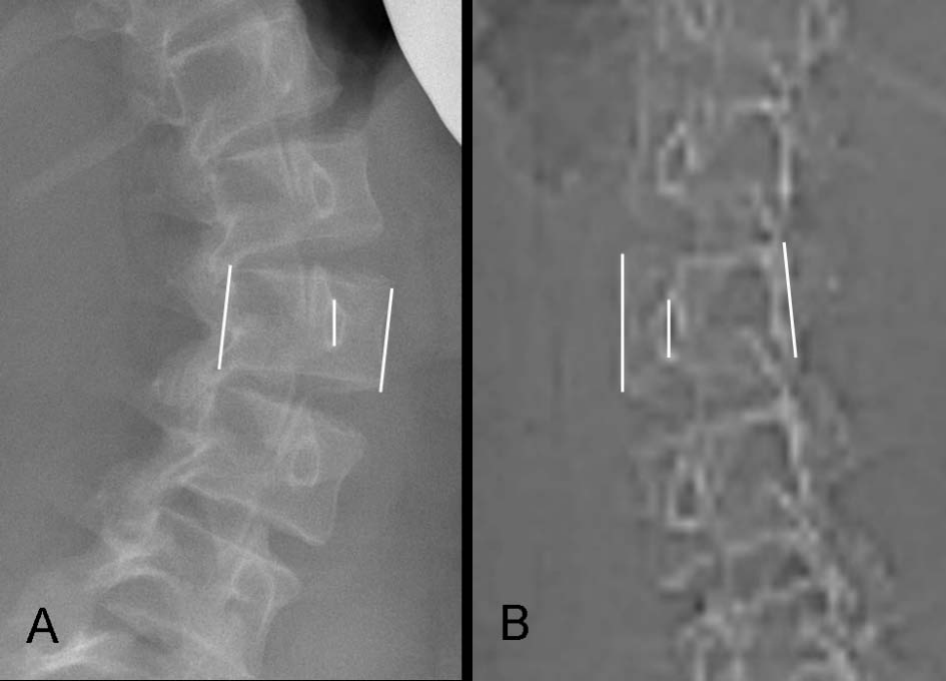
**(A) Standing plain radiograph, and (B) CT scanogram in prone position of a patient with AIS**. The vertebral rotation measured according to the method of Perdriolle at the apical vertebra of the major structural curve at L2 amounted to 43° and 25° on standing radiograph and CT scanogram, respectively.

**Figure 2:**
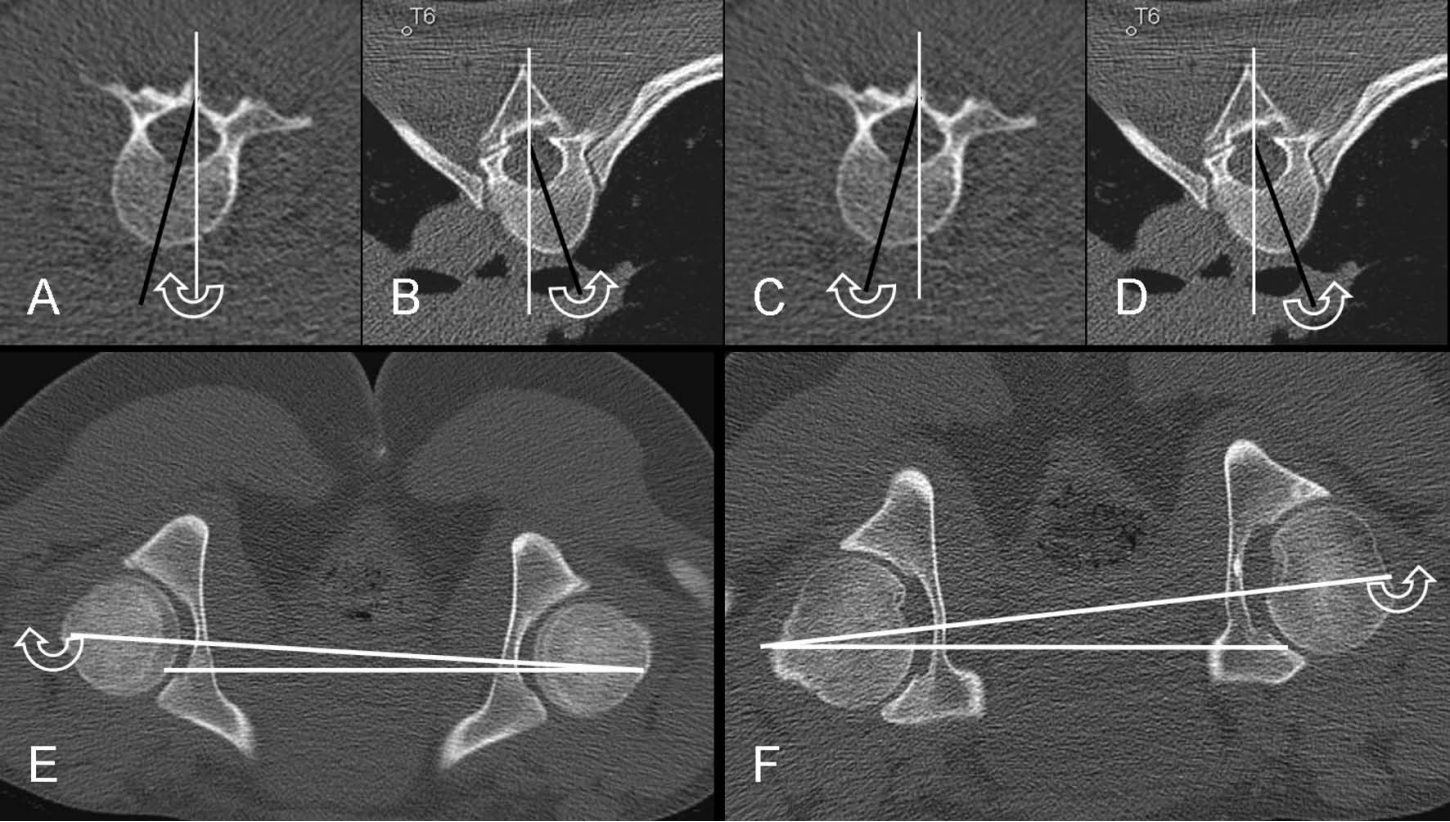
**CT with low radiation dose performed in prone position**. Axial images at the level of L2 (A and C), and at the level of T6 (B and D). (E-F) Axial images at the level of the femoral heads for the measurements of pelvic tilt. To obtain the corrected value of vertebral rotation, the degree of the pelvic tilt was subtracted from the measured degree of the rotation of the vertebral body when the vertebral body and the pelvis were tilted at the same direction (A/E and D/F). On the other hand, the degree of the pelvic tilt was added to the degree of the rotation of the vertebral body when the vertebral body and the pelvis were tilted at the different directions (B/E and C/F). Thus the corrected degree of vertebral rotation will be: A-E for example A, B+E in example B, C+F for example C, and D-F in example D.

Study analysis included the following radiological modalities and measurement methods:

(1) Standing frontal plain radiographs for the measurement of vertebral rotation according to the method of Perdriolle [2], Figure 1A.

(2) CT-scanograms in prone position for the measurement of vertebral rotation according to the method of Perdriolle [2]. Scanogram images were magnified to a size that was corresponding to the normal vertebral size and printed out for the purpose of measurement, (Figure 1B). The measurements of these examinations were compared with the measurements obtained by standing frontal plain radiograph (measurement No 1) for the evaluation of the impact of recumbent position on the vertebral rotation.

(3) CT in prone position for the measurement of vertebral rotation according to the method of Aaro and Dahlborn [9]: Axial 3 mm thick slices with skeletal algorithm and skeletal window were used for this purpose. The vertebral rotation was measured at the apical vertebra of the major structural curve and corrected to the pelvic tilt (Figure 2A-F).

(4) MRI in supine position for the measurement of vertebral rotation according to the method of Aaro and Dahlborn [9]. T1-weighted axial 3 mm thick images were used for this purpose. Comparison of these measurements with those obtained with CT in prone position (measurement No 3) enabled evaluation of the impact of the two different body positions (prone versus supine) on the degree of vertebral rotation.

All measurements were done by one reader (a senior radiologist; K.A.K) at two different occasions with 3-months interval. As low-dose CT has recently showed to be a reliable method in the evaluation of vertebral rotation [4], interobserver agreement was not the subject of analysis of this study.

### Statistical analysis

Statistical analysis was performed with SPSS 17 (originally; Statistical Package for the Social Sciences). Data is presented as proportions (%) or as mean with standard deviations (SD). Initially a linear regression analysis was done to compare the correlation between the measurements of vertebral rotation at two different occasions on axial CT images and on CT scanogram. The degree of intraobserver agreement with regard to the measurement of vertebral rotation on scanogram according to Perdriolle method and on axial CT images according to Aaro and Dahlborn method was evaluated by: (1) calculating a two-way mixed model of intraclass correlation coefficient (ICC), and (2) performing a paired sample t-test to calculate the systematic errors (mean value of differences), and the random errors (standard deviation of the differences). The interpretation of the ICC was done according to the one proposed by Landis [20]. A kappa value of 1 indicates a total agreement whereas a kappa of zero means poor agreement and indicates that any observed agreement is attributed to chance. Furthermore, the intraobserver agreement was quantified for the different methods of measurement using the approach recommended by Bland and Altman [18] calculating two descriptive statistics: the repeatability coefficient (√2SD^2^) and the coefficient of variation. The latter was calculated as a ratio of the repeatability coefficient over the mean value of the considered variables. Lower repeatability values and lower coefficient of variation mean better agreement between two measurements. Finally, Wilcoxon signed rank test was performed to compare the degree of vertebral rotation measured according to the two tested methods.

## Results

The mean ± SD value for Cobb angle was 56.6 ± 13.1° (median 55.6°, range 32.7-83°) and the mean value for pelvic tilt 2.4 ± 2° (median 2°, range 0.7°-8.2°). The mean value, SD and median value for the vertebral rotation estimated by the different methods are shown in Table 1. There was statistically significant difference when comparing the measurements of vertebral rotation done on CT scanogram with those done on axial CT images (mean 21.9°, and 17.4°, respectively; P = 0.02), Table 1.

**Table 1:** The mean values, standard deviation and median values (given in degrees) for the measurements of vertebral rotation according to different methods and in different body position.

Vertebral rotation	Mean	SD	Median
**Standing position**			
Radiographs, Perdriolle	25.7	9.8	26

**Recumbent, prone position**			
CT Scanogram, Perdriolle	21.9	8.3	21.5
Axial CT images, corrected to pelvis tilt, Aaro and Dahlborn	17.4	7.1	16.6
Axial CT images, not corrected to pelvis tilt, Aaro and Dahlborn	16.9	5.8	17.4

**Recumbent, supine position**			
MRI supine, Aaro and Dahlborn	16.1	6.5	15.1

The presented values are obtained from the two occasions of evaluation. The mean value for the pelvic tilt was 2.4° ± 2° (range 0.7°-8.2°).

### Reliability analysis

The intraobserver ICC was 0.83 for the measurements of vertebral rotation on prone CT scanogram according to the method of Perdriolle and 0.95 for those on axial CT images in prone position according to the method of Aaro and Dahlborn. There was good correlation on linear regression analysis with correlation coefficient of 0.69 for measurements of vertebral rotation on prone CT scanogram according to the method of Perdriolle and 0.91 for those on axial CT images in prone position according to the method of Aaro and Dahlborn, Table 2. However, the random error of the intraobserver differences (SD) was 5.1° for measurements on prone CT scanogram according to the method of Perdriolle and 2.3° for measurements on axial CT images in prone position according to the Aaro and Dahlborn method, Table 2. Furthermore, the repeatability coefficient and the coefficient of variation for measurements on the CT scanogram were 2.3 and 1.8 times higher than corresponding values for measurements on axial CT images (Table 2).

**Table 2:** The results of the reliability analysis of the measurements of vertebral rotation on standing plain radiograph and prone scanogram according to the method of Perdriolle, and on prone CT and supine MRI according to the method of Aaro and Dahlborn.

	Intraobserver reliability			
	**Plain Radiographs,** **(Perdriolle)**	**CT Scanogram, prone** **(Perdriolle)**	**CT, prone** **(Aaro and Dahlborn)**	**MRI, Supine** **(Aaro and Dahlborn)**

Systematic error (Mean)	1°	1.1°	0.2°	0.7°
Random error (SD)	7.1 °	5.1 °	2.3 °	3.1 °
Repeatability coefficient	10.2 °	7.2 °	3.2 °	4.4 °
Coefficient of variation	39.5%	32.8%	18.4%	27.3%
Intraclass correlation coefficient (ICC)	0.76	0.83	0.95	0.89
Correlation coefficient (r2) on linear regression analysis	0.61	0.69	0.91	0.81

### Influence of the method of measurement on the estimated degree of vertebral rotation

**Figure 3:**
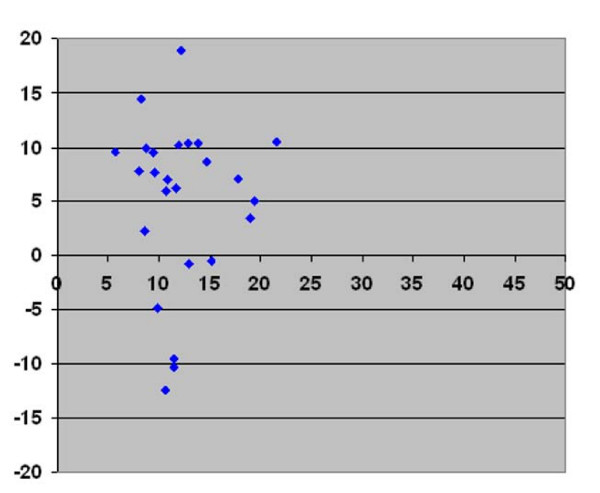
**CT scanogram versus axial CT images**. Plot diagram with Y-axis showing the difference between vertebral rotation measured on prone CT scanogram according to the Perdriolle method and vertebral rotation measured on axial CT images in prone position according to the Aaro and Dahlborn method against the X-axis showing the mean of the two measurements of vertebral rotation.

**Figure 4:**
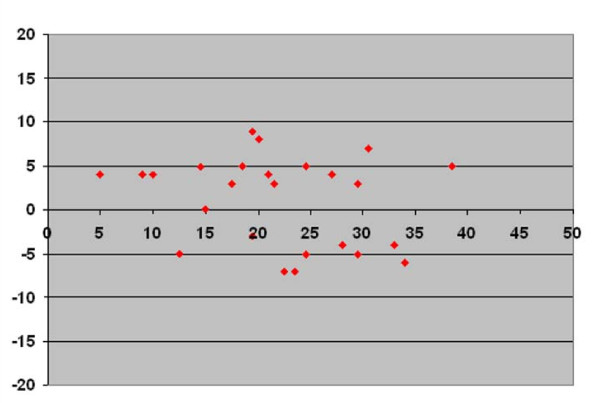
**CT scanogram**. Plot diagram with Y-axis showing the difference in vertebral rotation measured at two different occasions on the same CT scanograms in prone position according to the method of Perdriolle against the X-axis showing the mean of the two measurements of vertebral rotation measured on CT scanograms.

**Figure 5:**
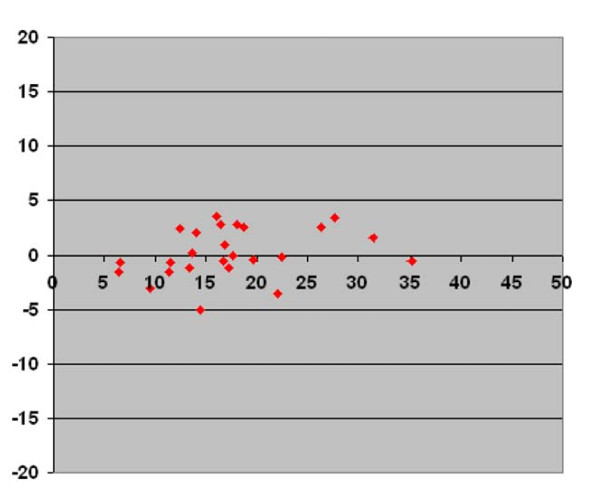
**Axial CT images**. Plot diagram with Y-axis showing the difference in vertebral rotation measured at two different occasions on the same axial CT images in prone position according to the method of Aaro and Dahlborn with values corrected to pelvic tilt against the X-axis showing the mean of the two measurements of vertebral rotation measured on axial CT images.

In Figures 3, 4, &5 we present the differences in vertebral rotation assessed by two different methods (Figure 3) and those assessed by the same method but measured at two different occasions (Figure 4, 5) in relation to the mean values of the vertebral rotation. The differences in all methods we calculated seemed to be independent of the severity of the rotation. When comparing the difference between estimated vertebral rotation assessed on CT scanograms and on axial CT images, there were 19 out of 25 patients that had a difference in the estimated rotation by the different methods of more than 5° (Figure 3). The corresponding values when assessing vertebral rotation by CT scanograms that was measured at two different occasions was 6 out of 25 patients (Figure 4). In contrast, the corresponding values when assessing vertebral rotation by the axial CT images that was measured at two different occasions was that none of the 25 patients had a difference in estimated vertebral rotation of more than 5° (Figure 5).

### Influence of body position on the estimated degree of vertebral rotation

When using the method of Perdriolle to compare the vertebral rotation in two different body positions, standing (plain radiograph)s versus prone (CT scanograms), vertebral rotation was reduced from 25.7 ± 9.8° in standing to 21.9 ± 8.3° in prone, a reduction of mean of 14.8% (P = 0.12). The magnitude of spontaneous correction varied from patients to patients with 11 out of 25 patients showed no correction or an increase in the degree of vertebral rotation on lying in prone position. Using the method of Aaro and Dahlborn to compare the vertebral rotation in prone position (axial CT images) with that in supine position (axial MRI), vertebral rotation was 16.9 ± 5.8° on axial CT images (before correction to the pelvic tilt) and 17.4 ± 7.1° (after correction to the pelvic tilt) compared to 16.1 ± 6.5° on axial MRI images, a difference of mean of 4.7% (p = 0.28) and 7.5% (p = 0.20), respectively, (Table 2).

## Discussion

This study has shown that the measurements of vertebral rotation according to the method of Aaro and Dahlborn on CT performed in prone position were more reliable than the measurements according to the method of Perdriolle performed on prone CT scanogram. Our study also showed that recumbent position compared to standing position in individuals with AIS achieved an almost 15% spontaneous correction of the vertebral rotation. Comparing the vertebral rotation on standing radiographs with that on scanogram (done in supine position) measured according to Perdriolle method, Yazici's et al [17] reported 24.4% spontaneous reduction of vertebral rotation compared with only 15% reduction reported in our study evaluated on scanogram in prone position. We believe that the discrepancy depends partly on lower reliability of the Perdriolle method (intraobserver SD of 5°), and partly on the fact that supine position causes higher degree of deformity correction than did the prone position, Table 2. The relatively lower image quality in CT scanogram compared with plain radiographs might have contributed to this discrepancy.

Regarding the impact of body position on the degree of vertebral rotation, the CT measurements in prone position (with and without correction to pelvic tilt) showed to achieve an average of 7.5% and 4.7%, respectively, lower correction than did the measurement on MRI in supine position. Based on this background, CT in prone position with measurements corrected to pelvic tilt used in this study has the following advantages: (a) lower radiation dose than plain radiography and other CT-protocols used in daily clinical practice (average effective radiation dose for low-dose CT was 0.37 mSv) [4], which in turn means lower risk for adverse effects caused by repeated radiation exposure, and (b) the method enables measurement of vertebral rotation before surgery in a position identical to the patient's position on the operation table. Furthermore, CT enables evaluation of the screw placement and deformity correction achieved by surgery as pedicle screws obscure pedicle shadow and make measurement of the postoperative vertebral rotation on standing plain radiography impossible. These evaluations are also difficult to perform on MRI as pedicle screws and rods give rise to disturbing susceptibility artifacts.

Our study also inferred that correction to pelvic tilt provide no more than in mean 1.3° difference between corrected and non corrected values. In addition, the reliability analysis showed that the random error of the intraobserver differences of measurements of vertebral rotation was 2.3° for CT in prone position and 3.1° for MRI in supine position. This indicates that the differences are so small that they are of no clinical significance when deciding the method of choice in the measurement of vertebral rotation. However, further studies should be conducted to study the impact of pelvic tilt correction on the estimation of vertebral rotation in patients with higher degrees of pelvic tilt and more pronounced vertebral rotations, as in patients with neuromuscular scoliosis, often associated with hip dysplasias and increased pelvic tilt.

## Conclusion

The measurement of vertebral rotation on axial CT images has shown to be more reliable than those on CT scanogram that correspond recumbent plain radiographs. Low-dose CT in prone position helps a correct screw insertion as it provides figures of vertebral rotation measured in a body position identical to that the surgeons usually are faced with on the operating table. Direct comparison between different methods of the measurement of vertebral rotation is questionable as both radiological modalities and body position influence the estimated degree of vertebral rotation. Vertebral rotation measured on CT in prone position and corrected to the pelvic tilt in patients with AIS imposes a low impact on the estimated degree of vertebral rotation than did measurements in supine position. However, the magnitude of differences between the measurements in these two body positions (prone versus supine) is of doubtful clinical significance in patients with AIS with moderate vertebral rotation and slight pelvis tilt.

## Competing interests

The authors declare that they have no competing interests.

## Authors' contributions

KAK has contributed to conception and design of the study, acquisition of data, analysis and interpretation of data, drafting the manuscript and has given his final approval of the version to be published.

MKK has contributed to interpretation of data, revision of the manuscript critically for important intellectual content, and has given his final approval of the version to be published.

RAH has contributed to revision of the manuscript critically for important intellectual content, and has given his final approval of the version to be published.

ACO has contributed to conception and design, interpretation of data, revising the manuscript critically for important intellectual content, and has given his final approval of the version to be published.
